# Serotype and virulence genes of Klebsiella pneumoniae isolated from mink and its pathogenesis in mice and mink

**DOI:** 10.1038/s41598-017-17681-8

**Published:** 2017-12-11

**Authors:** Wang Jian-li, Shang Yuan-yuan, Guo Shou-yu, Diao Fei-fei, Yu Jia-yu, Wei Xue-hua, Zhao Yong-feng, Jiang Shi-jin, Xie Zhi-jing

**Affiliations:** 1 Shandong Provincial Key Laboratory of Animal Biotechnology and Disease Control and Prevention, Shandong Agricultural University, Taian City, Shandong Province 271018 China; 2 College of Veterinary Medicine, Shandong Agricultural University, Taian City, Shandong Province 271018 China; 3 Shandong Provincial Engineering Technology Research Center of Animal Disease Control and Prevention, Shandong Agricultural University, Taian City, Shandong Province 271018 China

## Abstract

In the study, 15 K. pneumoniae strains were isolated from the mink experiencing respiratory distress in mideastern Shandong province, China, and the prevalence of K. pneumoniae in the sampled mink was 11.9% (15/126). Fourteen (93.33%) of the 15 K. pneumoniae isolates were identified as serotype K2 and hypermucoviscosity phenotype. The 12 virulence-associated genes of the K. pneumoniae isolates were tested. The prevalence of the wabG gene for the isolates were 100% (15/15), the ureA gene 100% (15/15), the rmpA gene 93.33% (14/15), the aerobactin gene 93.33% (14/15), the uge gene 93.33% (14/15), the IucB gene 80% (12/15) and the ybtA gene 13.33% (2/15). But the other five genes, fim, iroNB, wcaG, alls and kfuBC, gave a negative PCR reaction in the 15 isolates, respectively. The animal experiments using K. pneumoniae-SD-12 and K. pneumoniae-SD-21 demonstrated that the serotype K2 was high virulence for mice and mink. These finding implied there exist potential threat that K. pneumoniae pathogens could transmit to human, especially the fur animal farm workers and residents lived near the fur animal farms. Therefore, the etiology and epidemiological surveillance of K. pneumoniae in mink should be strengthened for people’s public health.

## Introduction

Klebsiella pneumoniae (K. pneumoniae), a member of the Enterobacteriaceae family, is an gram-negative bacillus causing hospital acquired infections and infections in debilitated or immunocompromised patients, such as hospital-acquired urinary tract infections, septicaemia pneumonia, pyogenic liver abscess (PLA) and metastatic complications^1–3^. The capsule is an important virulence factor, which protects K. pneumoniae from lethal serum factors and phagocytosis^4^. Alternately, as is the case in e.g. Streptococcus pneumoniae, capsular (K) types may be distributed across many unrelated clones due to frequent horizontal transfer of the capsular polysaccharide (CPS) operon, which is responsible for the synthesis of the capsular polysaccharide^5^. Among the 77 described K types of the serotyping scheme, serotypes K1, K2, K4 and K5 are highly virulent in experimental infection in mice and may cause severe infections in humans and animals^6,7^. And serotype K2 K. pneumoniae predominates in human infection^8^, which is the second most prevalent serotype next to serotype K1 as a cause of PLA and is also frequently reported in community acquired pneumonia^9^. The virulence of serotype K2 should not be underestimated^10^. And in French, a study of severe and fatal infections due to K. pneumoniae showed that the isolates from the fatal cases were all of capsular serotype K2^11^.

Identification of the specific bacterial virulence factors would help spur the development of rapid molecular diagnosis methods and innovative drug therapies^12^. Greater understanding of the virulence determinants of K. pneumoniae associated with liver abscess formation has focused on K serotypes and hypermucoviscosity phenotype, which is the invasive nature of certain K. pneumoniae isolates^13,14^. The other putative virulence factors have also been described, such as yersiniabactin (Ybt), aerobactin, and rmpA^13,15,16^. Ybt is a phenolate-type siderophore, which is structurally distinct from Ent^15^. And the aerobactin and rmpA genes have been identified to be simultaneously located on a 180-kilobase plasmid, and knockout of the rmpA gene can decrease virulence in mouse lethality tests by 1000-fold respectively^13,16^. Aerobactin, an iron chelator called iron siderophore, is an essential factor of pathogenicity in K. pneumoniae and can increase virulence in mouse lethality tests by 100-fold^16^. When injected into mice intraperitoneally, regardless of any K serotype, K. pneumoniae isolates with hypermucoviscosity phenotype as well as presence of rmpA and aerobactin genes exhibited high virulence for mouse lethality, 50% lethal dose (LD50) <10^2^ cell forming unit (CFU)^17^.

K. pneumoniae is responsible for a variety of diseases in humans and animals^18^. However, relatively few studies have specifically focused on mink. The objectives of the study were to clarify serotypes, hypermucoviscosity phenotype and virulence gene content of K. pneumoniae strains isolated from the mink showing respiratory distress in China. Furthermore, animal experiments were carried out to clarify whether experimental infection of mice and mink with the isolates resulted in clinical signs and pathological lesions.

## Results

### Serotypes and hypermucoviscosity phenotype of the K. pneumoniae isolates

In the study, 15 K. pneumoniae strains were isolated from the mink experiencing respiratory distress in mideastern Shandong province, China, named as K. pneumoniae-SD-1 to K. pneumoniae-SD-13, K. pneumoniae-SD-15 and K. pneumoniae-SD-21, and the prevalence of K. pneumoniae in the sampled mink was 11.9% (15/126). Fourteen (93.33%) of the 15 K. pneumoniae isolates belonged to serotype K2 using PCR and sequencing, and were identified as hypermucoviscosity phenotype by touching a colony with a loop and pulling up ≥5 mm, including K. pneumoniae-SD-1 to K. pneumoniae-SD-13 and K. pneumoniae-SD-21. However, K. pneumoniae-SD-15 was neither any of the serotypes nor hypermucoviscosity phenotype.

### Virulence-associated genes in the 15 K. pneumoniae isolates

The 12 virulence-associated genes of the K. pneumoniae isolates were tested using PCR and sequencing, and were shown in Table 1. The sequence analysis demonstrated that the prevalence of the wabG gene for the isolates was 100% (15/15), the ureA gene 100% (15/15), the rmpA gene 93.33% (14/15), the aerobactin gene 93.33% (14/15), the uge gene 93.33% (14/15), the IucB gene 80% (12/15) and the ybtA gene 13.33% (2/15). But the other five genes, fim, iroNB, wcaG, alls and kfuBC, gave a negative PCR reaction in the 15 isolates, respectively.

**Table 1:** The serotypes, HMV and virulence genes of the 15 K. pneumoniae isolates.

strains	K2	HMV	ybtA	ureA	IucB	rmpA	aerobactin	uge	wabG
KP-SD-1	+	+	−	+	+	+	+	+	+
KP-SD-2	+	+	−	+	+	+	+	+	+
KP-SD-3	+	+	−	+	+	+	+	−	+
KP-SD-4	+	+	−	+	+	+	+	+	+
KP-SD-5	+	+	−	+	+	+	+	+	+
KP-SD-6	+	+	−	+	+	+	+	+	+
KP-SD-7	+	+	−	+	+	+	+	+	+
KP-SD-8	+	+	−	+	+	+	+	+	+
KP-SD-9	+	+	−	+	+	+	+	+	+
KP-SD-10	+	+	−	+	+	+	+	+	+
KP-SD-11	+	+	−	+	+	+	+	+	+
KP-SD-12	+	+	+	+	−	+	+	+	+
KP-SD-13	+	+	+	+	−	+	+	+	+
KP-SD-15	−	−	−	+	−	−	−	+	+
KP-SD-21	+	+	−	+	+	+	+	+	+
P(%)	93.33	93.33	13.33	100	80	93.33	93.33	93.33	100

Note: KP, K. pneumoniae; HMV, hypermucoviscosity. +, positive; −, negative; P, prevalence.

### Pathogenesis of the K. pneumoniae isolates in mice

**Figure 1:**
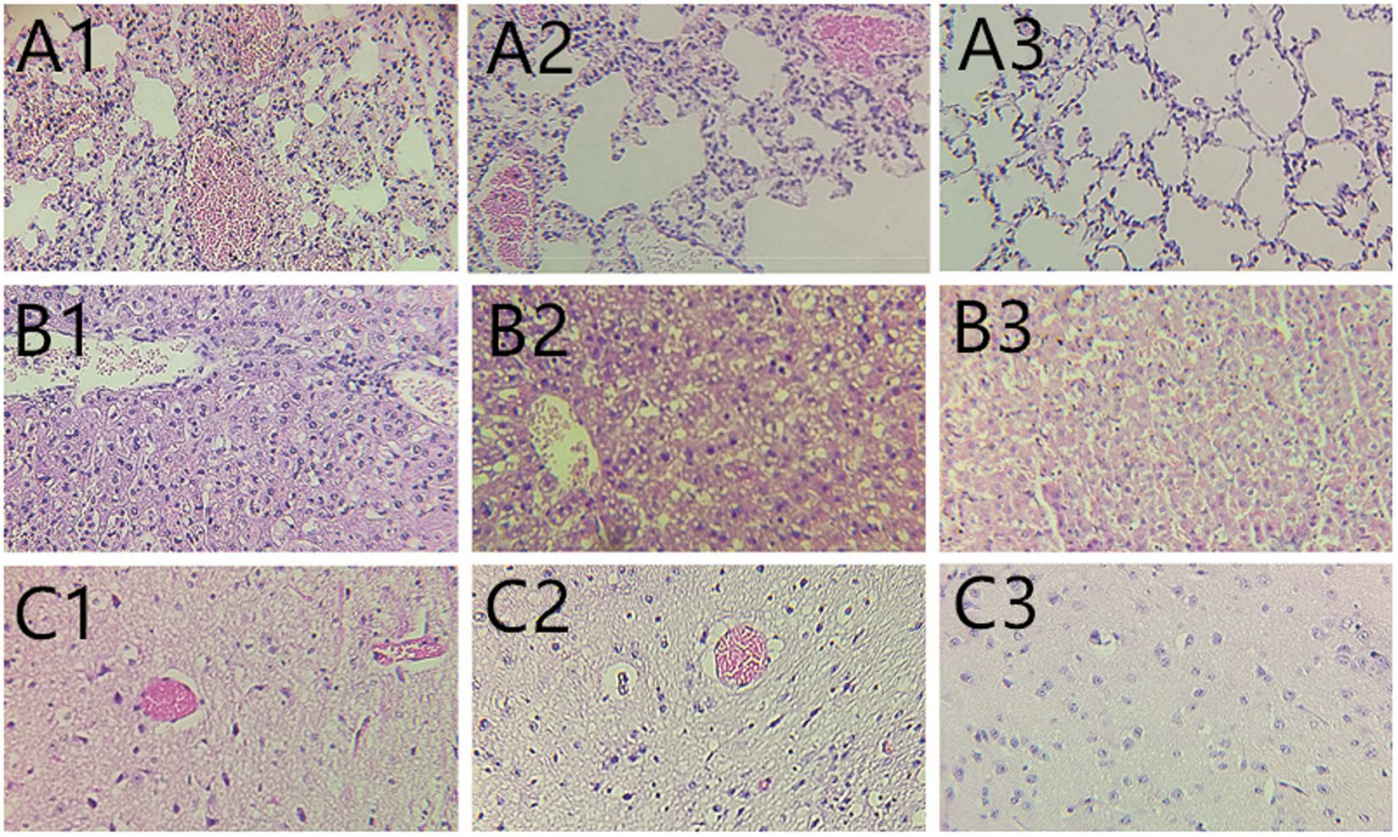
Histopathologic appearance of the tissues of the experimental mice. (**A1**) Lung tissue taken from a mouse died from K. pneumoniae-SD-12 infection on days 4 p.i., characterized by bleeding of the lung breakage. (**A2**) Lung tissue taken from a mouse died from K. pneumoniae-SD-21 infection on days 4 p.i., characterized by bleeding of the lung breakage. (**A3**) Lung tissue taken from a euthanized mouse inoculated with 0.9% NaCl solution on days 4 p.i. (**B1**) Liver tissue taken from a mouse died from K. pneumoniae-SD-12 infection on days 4 p.i., characterized by congesting and steatosis of the liver breakage. (**B2**) Liver tissue taken from a mouse died from K. pneumoniae-SD-21 infection on days 4 p.i., characterized by congesting and steatosis of the liver breakage. (**B3**) Liver tissue taken from a euthanized mouse inoculated with 0.9% NaCl solution on days 4 p.i. (**C1**) Brain tissue taken from a mouse died from K. pneumoniae-SD-12 infection on days 4 p.i., characterized by light bleeding and edema of the brain breakage. (**C2**) Brain tissue taken from a mouse died from K. pneumoniae-SD-21 infection on days 4 p.i., characterized by light bleeding and edema of the brain breakage. (**C3**) Brain tissue taken from a euthanized mouse inoculated with 0.9% NaCl solution on days 4 p.i. HE stain. Original magnification was × 200 for all images.

In 30 h postinfection (p.i.), most of the mice in the groups inoculated intraperitoneally with 10^8.0^ CFU and 10^9.0^ CFU using K. pneumoniae-SD-12, K. pneumoniae-SD-15 and K. pneumoniae-SD-21 died without definite clinical signs and histopathology changes, but abdominal cavity liquid of the mice inoculated with K. pneumoniae-SD-12 and K. pneumoniae-SD-21 pulled up ≥5 mm. On days 2–6 p.i, all the other challenged mice gradually showed clinical designs, including partial loss of appetite, coarse fur, sneezing and coughing. Some of the animals died from K. pneumoniae infection, which reached a peak on days 4–5 p.i. The dead mice showed lung hemorrhage, liver hemorrhage and swelling, slight bleeding point in brain, but no liver abscess. Compared to the control mice, histologic lesions were found in the mice that died from K. pneumoniae infection, such as lung bleeding and congesting, liver congesting and steatosis, and light bleeding and edema in brain tissues (Fig. 1). The survived mice were debilitated, but resumed eating and achieved complete clinical recovery. The LD50 of K. pneumoniae-SD-12 in mice was 5.0 × 10^2.0^ CFU, the LD50 of K. pneumoniae-SD-15 3.2 × 10^8.0^ CFU, and the LD50 of K. pneumoniae-SD-21 2.0 × 10° CFU. The virulence of K. pneumoniae-SD-21 in mice was higher than that of K. pneumoniae-SD-12. The control mice showed no clinical signs.

### Pathogenesis of the K. pneumoniae isolates in mink

**Figure 2:**
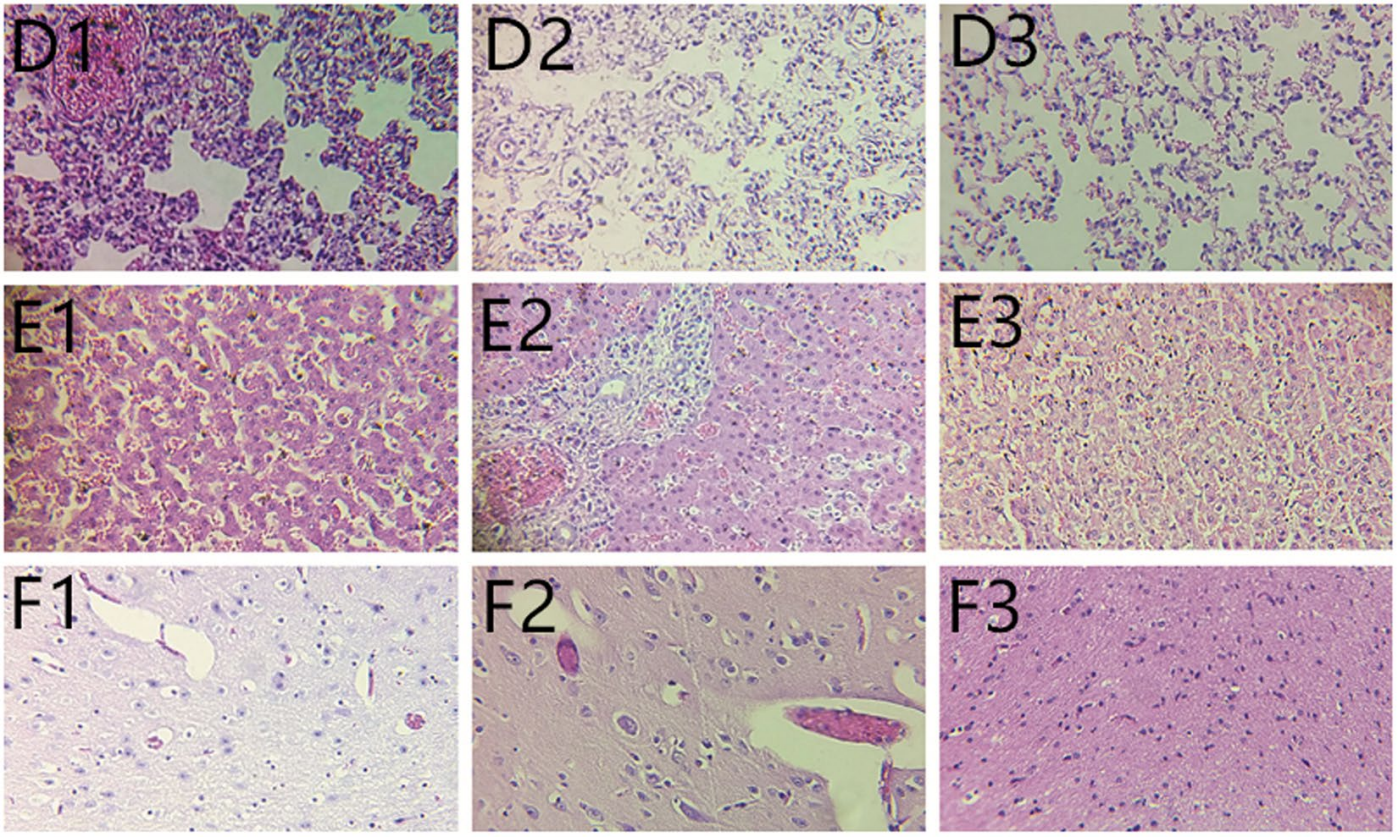
Histopathologic appearance of the tissues of the experimental mink. (**D1**) Lung tissue taken from a mink died from K. pneumoniae-SD-12 infection on days 5 p.i., characterized by bleeding and congesting of the lung breakage. (**D2**) Lung tissue taken from a mink died from K. pneumoniae-SD-21 infection on days 5 p.i., characterized by bleeding of the lung breakage. (**D3**) Lung tissue from a euthanized mink inoculated with 0.9% NaCl solution on days 5 p.i. (**E1**) Liver tissue taken from a mink died from K. pneumoniae-SD-12 infection on days 5 p.i., characterized by congesting and steatosis of the liver breakage. (**E2**) Liver tissue taken from a mink died from K. pneumoniae-SD-21 infection on days 5 p.i., characterized by congesting and steatosis of the liver breakage. (**E3**) Liver tissue from a euthanized mink inoculated with 0.9% NaCl solution on days 5 p.i. (**F1**) Brain tissue taken from a mink died from K. pneumoniae-SD-12 infection on days 5 p.i., characterized by light bleeding and edema of the brain breakage. (**F2**) Brain tissue taken from a mink died from K. pneumoniae-SD-21 infection on days 5 p.i., characterized by light bleeding and edema of the brain breakage. (**F3**) Brain tissue from a euthanized mink inoculated with 0.9% NaCl solution on days 5 p.i. HE stain. Original magnification was × 200 for all images.

On days 2–8 p.i., some of the challenged mink showed clinical signs, including partial loss of appetite, coarse fur, sneezing and coughing. Some of the mink died from K. pneumoniae infection, which reached a peak on days 5–6 p.i. The dead mink showed lung hemorrhage, liver hemorrhage and swelling, and slight bleeding point in brain, but no liver abscess. Compared to the control mink, histologic lesions were found in the inoculated mink, such as lung bleeding and congesting, liver congesting and steatosis, pulling up ≥5 mm of abdominal cavity liquid, light bleeding and edema in brain tissues (Fig. 2). The survived mink were debilitated, but resumed eating and achieved complete clinical recovery. The LD50 of K. pneumoniae-SD-12 in mink was 1.3 × 10^3.0^ CFU, the LD50 of K. pneumoniae-SD-15 8.0 × 10^8.0^ CFU, and the LD50 of K. pneumoniae-SD-21 3.2 × 10^1.0^ CFU. The virulence of K. pneumoniae-SD-21 in mink was higher than that of K. pneumoniae-SD-12. The control mink showed no clinical signs.

## Discussion

K. pneumoniae is found in the environment and as a harmless commensal, but is also a frequent nosocomial pathogen causing urinary, respiratory and blood infections, and PLA^19–21^. The K serotype, lipopolysaccharide and iron scavenging systems play an important role in the virulence of K. pneumoniae^22^. The K serotypes and hypermucoviscosity phenotype are the invasive nature of certain K. pneumoniae strains^13,14^. Serotypes K1, K2, K4 and K5 are highly virulent in experimental infection in mice and may cause severe infections in humans and animals^4,6,7^. And serotype K2 K. pneumoniae predominates in human infection^8,23,24^, which is the second most prevalent serotype next to serotype K1 as a cause of PLA and is also frequently reported in community acquired pneumonia^9^. In the study, 14 (93.33%) of the 15 K. pneumoniae isolates were identified as serotype K2 and hypermucoviscosity phenotype. It implied that serotype K2 was prevalent in mink in China.

The other putative virulence factors have also been described, such as Ybt, aerobactin, and rmpA^13,15,16^. The rmpA-carrying strains were associated with the hypermucoviscosity phenotype and the invasive clinical syndrome^12,14^. Aerobactin supplementation of a defined minimal medium with transferrin markedly reduced the growth of avirulent strains but had no significant effect on the growth of virulent strains, and production of aerobactin could be correlated with virulence^16^. In this study, K. pneumoniae-SD-12 and K. pneumoniae-SD-21 were positive for rmpA and aerobactin genes, and showed high virulent to mice and mink (LD50 less than 10^3.0^ CFU). Furthermore, serotype K2 and hypermucoviscosity phenotype should contribute to enhance virulence of K. pneumoniae-SD-12 and K. pneumoniae-SD-21 in mice and mink. The virulence gene content difference influenced virulence of K. pneumoniae^19^. The relatively higher virulence of K. pneumoniae-SD-21 in mice and mink than that of K. pneumoniae-SD-12, might be partly due to the virulence gene content difference between K. pneumoniae-SD-12 and K. pneumoniae-SD-21 (Table 2). But the definite mechanism need to be further studied. K. pneumoniae-SD-15, containing uge, wabG and ureA genes, was avirulent to mice and mink (LD50 more than 10^8.0^ CFU). The K. pneumoniae uge mutants were unable to produce experimental urinary tract infections in rats and were completely avirulent in two different animal models (septicemia and pneumonia)^25^. K. pneumoniae waaC, waaF, and wabG mutants were avirulent when tested in different animal models^26^.

**Table 2:** Specific primers used for amplification of the target genes of K. pneumoniae.

Target	Primer	Sequence (5′–3′)	Product size (bp)	Reference
Capsular type K1	MagAF1	GGTGCTCTTTACATCATTGC	1283	^9^
	MagAR1	GCAATGGCCATTTGCGTTAG		
Capsular type K2	K2wzyF1	GACCCGATATTCATACTTGACAGAG	641	^9^
	K2wzyR1	CCTGAAGTAAAATCGTAAATAGATGGC		
Capsular type K5	K5wzxF	TGGTAGTGATGCTCGCGA	280	^9^
	K5wzxR	CCTGAACCCACCCCAATC		
Capsular type K20	wzyK20F	CGGTGCTACAGTGCATCATT	741	^7^
	wzyK20R	GTTATACGATGCTCAGTCGC		
Capsular type K54	wzxK54F	CATTAGCTCAGTGGTTGGCT	881	^7^
	wzxK54R	GCTTGACAAACACCATAGCAG		
Capsular type K57	wzyK57F	CTCAGGGCTAGAAGTGTCAT	1037	^7^
	wzyK57R	CACTAACCCAGAAAGTCGAG		
rmpA	rmpAF	ACTGGGCTACCTCTGCTTCA	536	^9^
	rmpAR	CTTGCATGAGCCATCTTTCA		
Aerobactin	aerobactinF	GCATAGGCGGATACGAACAT	556	^9^
	aerobactinR	CACAGGGCAATTGCTTACCT		
Alls	allsF	CCGAAACATTACGCACCTTT	1090	^9^
	allsR	ATCACGAAGAGCCAGGTCAC		
kfuBC	kfuBC-F	GAAGTGACGCTGTTTCTGGC	797	^29^
	kfuBC-R	TTTCGTGTGGCCAGTGACTC		
wcaG	wcaG-F	GGTTGGKTCAGCAATCGTA	169	^7^
	wcaG-R	ACTATTCCGCCAACTTTTGC		
IucB	iucB-F	ATGTCTAAGGCAAACATCGT	948	^29^
	iucB-R	TTACAGACCGACCTCCGTGA		
iroNB	iroNB-F	GGCTACTGATACTTGACTATTC	992	^29^
	iroNB-R	CAGGATACAATAGCCCATAG		
ureA	ureA-F	GCTGACTTAAGAGAACGTTATG	337	^30^
	urea-R	GATCATGGCGCTACCT(C/T)A		
wabG	wabG-F	CGGACTGGCAGATCCATATC	683	^31^
	wabG-R	ACCATCGGCCATTTGATAGA		
uge	uge-F	GATCATCCGGTCTCCCTGTA	535	^31^
	uge-R	TCTTCACGCCTTCCTTCACT		
fim	fim-F	TGCTGCTGGGCTGGTCGATG	550	^31^
	fim-R	GGGAGGGTGACGGTGACATC		
ybtA	ybtA-F	ATGACGGAGTCACCGCAAAC	960	^29^
	ybtA-R	TTACATCACGCGTTTAAAGG		

It was the first to identify that serotype K2 K. pneumonia was prevalent in mink in China. Based on the animal experiments, K. pneumoniae-SD-12 and K. pneumoniae-SD-21 showed high virulent to mice and mink, and the K2 infection did cause diseases in mice and mink. Our findings suggest that the potential exists for K. pneumoniae transmission to humans, especially the fur animal farm workers and residents lived near the fur animal farms. Therefore, the etiology and epidemiological surveillance of K. pneumoniae in mink should be strengthened for people’s public health.

## Materials and Methods

### K. pneumoniae isolation

During April 2014 to May 2015, 126 lung samples of the mink experiencing respiratory distress were collected in mideastern Shandong province, China. The K. pneumoniae strains were isolated from the samples according to standard clinical microbiologic methods. After inoculation on nutrient agar plates and incubation at 37 °C overnight, the string test was performed by touching a colony with a loop and pulling up. A test result is considered to be positive when a string of ≥5 mm is observed^27^. A bacterial colony from an overnight culture was added to 300 μL water and boiled for 15 min to release DNA template^9^. The isolates were identified using PCR based on the khe gene, a specific target gene of K. pneumonia, and the specific primers were 5′-TGATTGCATTCGCCACTGG-3′ and 5′- GGTCAACCCAACGATCCTG-3′, and the length of expected PCR products is 486 bp as described previously^28^.

### Serotype and virulence-associated gene detection

A bacterial colony from an overnight culture was added to 300 μL water and boiled for 15 min to release DNA template^9^. The isolates were serotyped using PCR for serotypes K1, K2, K5, K20, K54 and K57, and 12 virulence-associated genes in the isolates were screened using PCR as described previously, including rmpA, aerobactin, wcaG, ybtA, iucB, iroNB, ureA, uge, kfuBC, fim, wcaG and allS genes^7,9,29–31^. The specific primers and the length of expected PCR products were shown in Table 2. The PCR conditions used were available upon request. The PCR products were extracted from agarose gels, using a GenScript QuickClean gel extraction kit (GenScript, Piscataway, NJ, USA), and sequencing was performed in Sangon Biological (Shanghai) Co., Ltd (Shanghai, China). The nucleotide sequences of the corresponding genes of the isolates were submitted to the GenBank, and were assigned GenBank accession numbers KY403895-KY403994. All nucleotide sequence data were edited by the Lasergene sequence analysis software package (DNASTAR, Madison, WI, USA). BLAST analyses were used on each sequence to identify the related reference isolates. The nucleotide sequences were compared with MEGA6.0 using Clustal W.

### Pathogenesis of the K. pneumoniae isolates in mice

To clarify the pathogenicity of the K. pneumoniae isolates in mice, the experiments were performed on 165 healthy Kunming mice (aged 6 to 8 weeks), which were divided into 33 groups on average (5 mice per group). According to serotypes, hypermucoviscosity phenotype and virulence gene content of K. pneumoniae isolates, K. pneumoniae-SD-12, K. pneumoniae-SD-15 and K. pneumoniae-SD-21 were selected for animal experiments. The K. pneumoniae isolates were individually incubated overnight at 37 °C. Bacterial concentration was calculated by CFU. Just prior to use, the microorganisms forming smooth mucoid colonies were selected and 10-fold serial dilutions with 0.9% of endotoxin-free normal saline. The mice in the 1–10 groups were lightly anesthetized with ketamine chloride by intramuscular injection and were intraperitoneally inoculated with 10^9.0^ CFU, 10^8.0^ CFU, 10^7.0^ CFU, 10^6.0^ CFU, 10^5.0^ CFU, 10^4.0^ CFU, 10^3.0^ CFU, 10^2.0^ CFU, 10^1.0^ CFU and 10° CFU_,_ respectively, using K. pneumoniae-SD-12, the mice in the 12–21 groups using K. pneumoniae-SD-15 and the mice in the 23–32 groups using K. pneumoniae-SD-21. The mice in Group 11, 22 and 33 were inoculated intraperitoneally with 0.9% NaCl solution, serving as the control group, respectively. The animals were individually housed. Commercial qualified food and water were freely available at all times.

From postinfection (p.i.) onwards, clinical signs of the mice were monitored and scored daily for 15 days or until the inoculated mice died from K. pneumoniae infection. The tissue samples were collected from the mice either killed by K. pneumoniae infection or euthanized on days 15 after K. pneumoniae inoculation, including cerebrum, cerebellum, lung, myocardium, liver, spleen and kidney. The samples were rapidly immersed in 10% neutral formalin buffer to prevent autolysis, and then processed into paraffin, sectioned at 4 μm using the microtome Leica RM2235 (Leica Microsystems Ltd.), and stained with hematoxylin and eosin (HE) for the detection of histological lesions by light microscopy. The LD50 of K. pneumoniae in mice was titrated using Reed and Muench^32^. The degree of virulence was read as highly virulent for an LD50 of ≤10^3.0^ CFU, moderate virulence for an LD50 of 10^4.0^–10^5.0^ CFU, low virulence for an LD50 of 10^6.0^–10^7.0^ CFU, and no virulence for an LD50 of ≥ 10^8.0^ CFU^33^.

### Pathogenesis of the K. pneumoniae isolates in mink

To clarify the pathogenicity of the K. pneumoniae isolates in mink, the animal experiments were performed on 90 healthy American mink (2 months of age), which were divided into 18 groups on average. The mink in the 1–5 groups were lightly anesthetized with ketamine chloride by intramuscular injection and were intraperitoneally inoculated with 10^5.0^ CFU, 10^4.0^ CFU, 10^3.0^ CFU, 10^2.0^ CFU and 10^1.0^ CFU, respectively, using K. pneumoniae-SD-12, and the mink in the 7–11 groups using K. pneumoniae-SD-21. The mink in 13–17 groups were intraperitoneally inoculated with 10^9.0^ CFU, 10^8.0^ CFU, 10^7.0^ CFU, 10^6.0^ CFU and 10^5.0^ CFU respectively, using K. pneumoniae-SD-15. The mink in Group 6, 12 and 18 were inoculated intraperitoneally with 0.9% NaCl solution, serving as the control group, respectively. The animals were housed individually and fed twice daily on a commercial meat-based diet. Water was freely available at all times.

From postinfection (p.i.) onwards, clinical signs of the mink were monitored and scored daily for 15 days or until the inoculated mink died from K. pneumoniae infection. The tissue samples were collected from the mink either killed by K. pneumoniae infection or euthanized on days 15 after K. pneumoniae inoculation, including cerebrum, cerebellum, lung, myocardium, liver, spleen and kidney. The samples were rapidly immersed in 10% neutral formalin buffer to prevent autolysis, and then processed into paraffin, sectioned at 4 μm using the microtome Leica RM2235 (Leica Microsystems Ltd.), and stained with HE for the detection of histological lesions by light microscopy. The LD50 of K. pneumoniae in mink was titrated using Reed and Muench^32^.

### Ethics Statement

All animal experiments were performed in accordance with regulatory standards and guidelines approved by the Shandong Agricultural University’s Animal Care and Use Committee, and the approved is NO. SDAUA-2015-010.
